# Ants improve the reproduction of inferior morphs to maintain a polymorphism in symbiont aphids

**DOI:** 10.1038/s41598-018-20159-w

**Published:** 2018-02-02

**Authors:** Saori Watanabe, Jin Yoshimura, Eisuke Hasegawa

**Affiliations:** 1 Laboratory of Animal Ecology, Department of Ecology and Systematics, Graduate School of Agriculture, Hokkaido University, Kita-ku, Kita-9-Nishi-9, Sapporo, 060-8589 Japan; 2 Graduate School of Science and Technology and Department of Mathematical and Systems Engineering, Shizuoka University, 3-5-1 Johoku, Naka-ku, Hamamatsu, 432-8561 Japan; 3 Marine Biosystems Research Center, Chiba University, Uchiura, Kamogawa, Chiba 299-5502 Japan; 4 Department of Environmental and Forest Biology, State University of New York College of Environmental Science and Forestry, Syracuse, NY 13210 USA

## Abstract

Identifying stable polymorphisms is essential for understanding biodiversity. Distinctive polymorphisms are rare in nature because a superior morph should dominate a population. In addition to the three known mechanisms for polymorphism persistence, we recently reported a fourth mechanism: protection of the polymorphism by symbionts. Attending ants preferentially protect polymorphic aphid colonies consisting of green and red morphs. Here, we show that attending ants manipulate the reproductive rate of their preferred green morphs to equal that of the red morphs, leading to the persistence of the polymorphism within the colonies. We could not, however, explain how the ants maintained the polymorphism in aphid colonies regardless of inter-morph competition. Manipulation by symbionts may be important for the maintenance of polymorphisms and the resulting biodiversity in certain symbiotic systems.

## Introduction

Many natural ecosystems exhibit fairly high biodiversity, and the reason for such high biodiversity is the most fundamental question in ecology and evolutionary biology^1^ because interspecific competition excludes competing species^2^. However, natural communities consist of many competing species. A heritable genetic polymorphism is one such form of biodiversity for which persistence is difficult to explain. In this sense, the persistence of a polymorphism is a fundamentally important issue in ecology and evolution because it helps us understand the observed biodiversity in nature.

Why does a polymorphism persist in a population? Previously, three major mechanisms were identified: (1) negative frequency-dependent selection, (2) balancing selection of two opposing factors, and (3) superdominance. Under negative frequency-dependence, any morph becomes advantageous when rare but disadvantageous when abundant^3^. Therefore, polymorphisms are stable because each morph is protected from extirpation. However, polymorphisms caused by two opposing factors are rather unstable because random processes (genetic drift) can easily lead to the extinction of any one morph. Therefore, an averaging mechanism that protects from random walk is necessary in these polymorphisms (e.g., spatial heterogeneity that yields patchy microhabitats)^4^. Superdominance is the phonemenon that the heterozygote shows a higher fitness than the homozygotes, and thus two alleles are maintained stably in a population.

**Figure 1:**
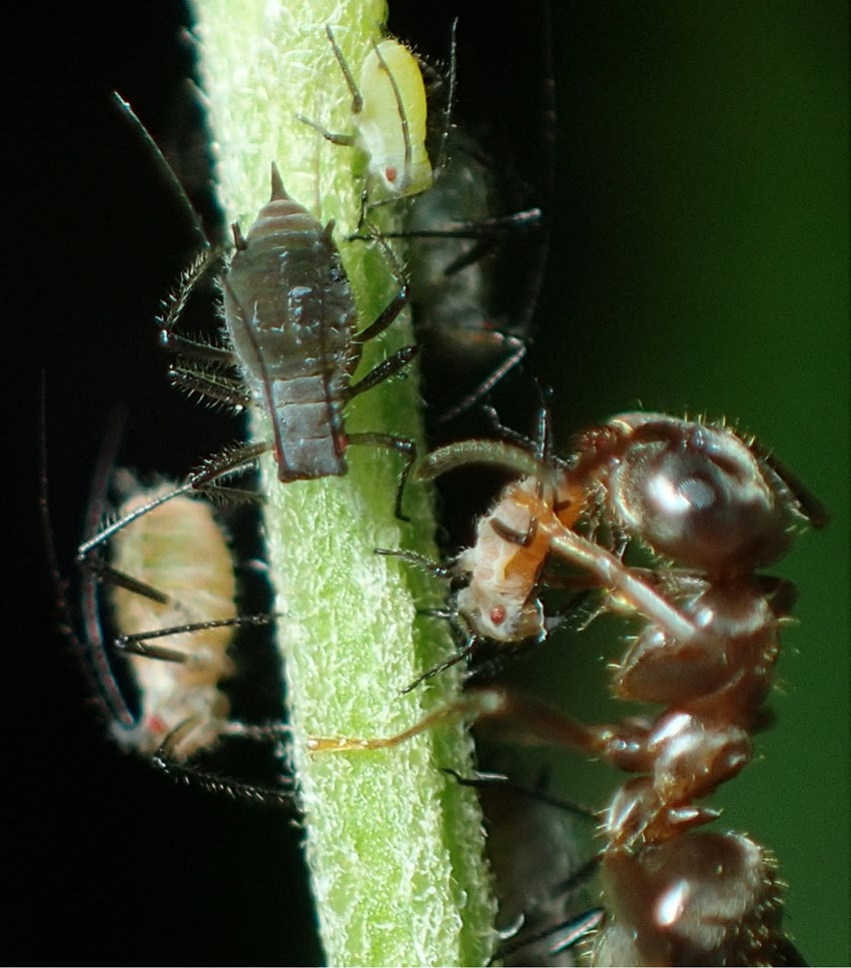
Red and green morphs of *M. yomogicola* with the attending ant *L. japonicus*. The top three aphids (small green and two large black) are green morphs, and the lower two aphids (small brown with an ant and a large brownish green) are red morphs. Photo by Ryota Kawauchiya.

Recently, we found another mechanism for the maintenance of color polymorphism in an ant-aphid symbiotic system. The aphid *Macrosiphoniella yomogicola* has established an obligate symbiotic relationship with ants that promotes the survival of the aphid because the attending ants provide protection from strong predation^5^. *M. yomogicola* exhibits a heritable color polymorphism with green and red morphs (Fig. 1)^5^. We found that the attending ant *Lasius japonicus* (Fig. 1) is attracted to and most strongly guards a mixed aphid colony (approximately 65% green morph)^5^. Because *L. japonicus* most strongly protects the mixed aphid colonies, the color polymorphism in the aphid is maintained in a population. In fact, a previous study has shown that polymorphic aphid colonies showed lower mortality^6^. However, a stable polymorphism is not guaranteed in this symbiotic system. For example, if the morphs differ in competitive ability or growth rate, any aphid colony will soon lose the inferior morph. Here, we use the term “competitive ability” in a broad sense, and it includes differences in the rates of growth and reproduction even if actual competitive interactions (e.g., depriving a resource from the other morph) do not occur between the morphs. Note that any such difference between morphs leads to the exclusion of the inferior morphs. Surprisingly, nearly all colonies consist of both green and red morphs in the field. However, the mechanism that maintains these two morphs in a colony when the morphs present different reproductive rates has not been clarified.

Ant-attended aphids are known to excrete high-quality honeydew when ants are present^7,8^. Ant attendance has a negative effect on the growth and reproduction of the attended aphids^9^. Therefore, trade-offs should occur between the quality of honeydew and the growth and fecundity of aphid individuals. Thus, if attending ants prefer the morph excreting a high-quality honeydew, such trade-offs and resulting competitive interactions are expected between the color morphs in *M. yomogicola*. The morph excreting high-quality honeydew is known to have a lower reproductive rate than the other morphs^9,10^. This fact implies that if the attending ants prefer one morph, this morph is expected to excrete high-quality honeydew. Note that honeydew quality is known to vary between preferred and non-preferred morphs^11^. Therefore, the preferred morph is expected to decrease in number over time and eventually disappear from a colony, even if the ants prefer them. Regardless of this inferior trait, the morph that is preferred by ants is more likely to survive from spring to autumn when *M. yomogicola* reproduces asexually and to late autumn when the aphid produces sexuparae. The mated sexual females lay eggs that will overwinter. Thus, a strong attractiveness to ants is important for an aphid clone.

However, in our field, the coexistence of the two color morphs on every host plant continues from June (the appearances of alates) to November (the end of the season). How can the two color morphs achieve long-lasting coexistence on the same host plant when the competitive exclusion of the inferior morphs is expected?

This study aims to understand the long-lasting coexistence of both color morphs in every colony of *M. yomogicola* on host plants. In this study, we investigated the population growth and parthenogenetic reproductive rates of both morphs of *M. yomogicola* under several experimental settings. First, we compared the reproductive rate of each monoclonal red morph and green morph on several cloned host plants. Second, we examined the difference in the reproductive rates between the color morphs using a single cloned host plant. Then, we measured the reproductive rate of each morph in mixed colonies free from predators with or without attending ants. Finally, we tested the ants’ preference for the color morphs. The results showed that attending ants improved the reproductive rate of their preferred green morph, and without attending ants, the green morphs had a lower reproductive rate than the red morphs. Because of the ants’ manipulation, the reproductive rate of the green morphs is equal to that of the red morphs. This manipulation enables an aphid colony to maintain a long-lasting coexistence of both morphs in a single colony on a host plant.

## Results

**Figure 2:**
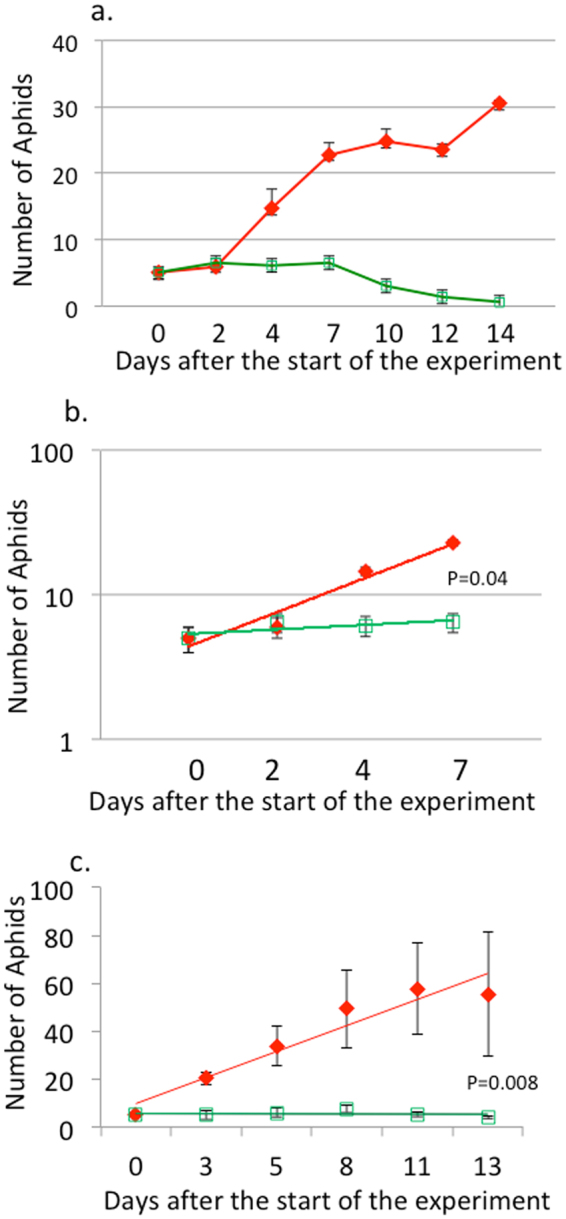
Reproductive rates of both red and green morphs of *M. yomogicola* without ant attendance. (**a**) Changes in the number of five different clones of green and red morphs reared on a single clone of the host plant. After seven days, both morphs decreased because of environmental deterioration caused by their own excreted honeydew. (**b**) Linear regressions of the log-transformed aphid numbers over time for each morph (the marks are the average of the 5 clones) on a single host plant clone for the first 4 records (7 days) in Fig. 2a. The slope is steeper for the red morph than for the green morph (ANCOVA, t = 2.733, n = 6, P = 0.009), indicating that the red morph increased more rapidly than the green morph on the same host plant. (**c**) Linear regression of aphid numbers over time for a single clone of both color morphs on five different clones of the host plant. The slope is significantly steeper for the red morph than for the green morph (ANCOVA, t = −2.741, n = 5, P = 0.008), showing that the red clone increased more rapidly than the green morph, irrespective of variations in the host plant clones.

Five clones each of both morphs were reared on one clonal shoot of a single host plant (mugwort: *Artemisia vulgaris*). Without attending ants, the number of aphids increased quickly (7 days) but were soon saturated, and they then decreased because of the environmental deterioration caused by their own excreted honeydew (Fig. 2a). This trend was more striking for the green morphs than for the red morphs (Fig. 2a). We then compared the reproductive rates of both morphs for the first 4 records (7 days) that were not affected by the environmental deterioration. Model selection using the AIC provided a simple linear regression with log-transformed aphid numbers as the best model (Supplementary Table S1). The regression showed that the reproductive rate of red clones was significantly higher than that of green clones (Fig. 2b). Then, we compared the reproductive rates of a red and a green clone on 5 different clones of the host plant. Again, on average, the reproductive rate of the red morphs was significantly higher than that of the green morphs on the five host plant clones (Fig. 2c). Thus, the reproductive rate of the red morphs was significantly higher than that of the green morphs, irrespective of the difference in aphid clones (Fig. 2a and b) or host plant clones (Fig. 2c).

**Figure 3:**
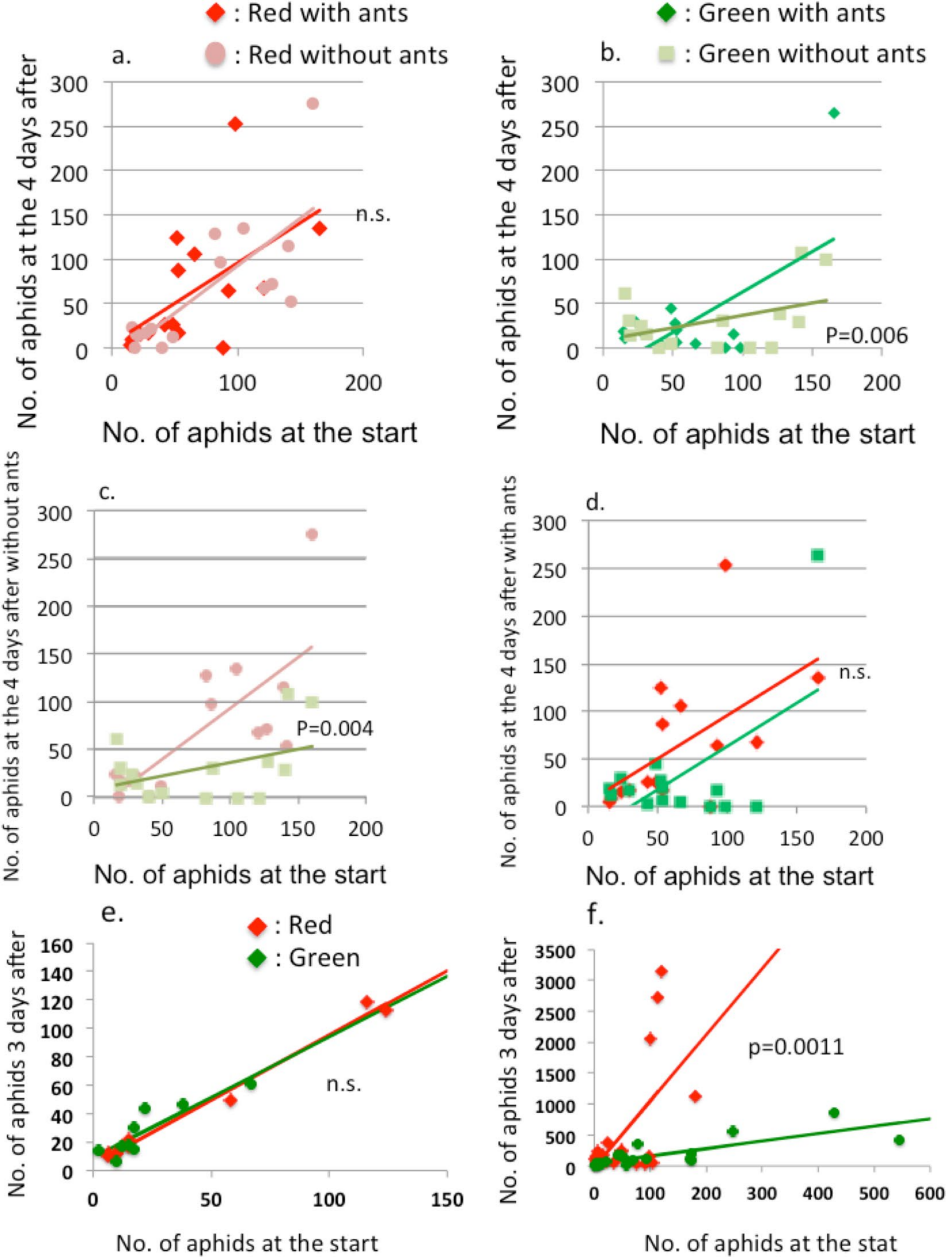
Patterns in reproductive rates of both green and red morphs in mixed-color colonies in the field. (**a**) Reproductive rates of the red morphs with or without attending ants. No significant differences in the reproductive rates were detected with or without ants (ANCOVA, *F* = 0.2049, df = 1, 26, p = 0.6546). (**b**) Reproductive rates of the green morphs with or without attending ants. The green morphs with attending ants increased significantly faster than those without ants (ANCOVA, *F* = 9.5709, df = 1, 26, p = 0.006). (**c**) Under ant-absent conditions, the red morph showed a significantly faster increase than did the green morph (ANCOVA, *F* = 3.094, df = 1, 26, p = 0.004); (**d**) however, this difference disappeared when ants were present (ANCOVA, *F* = 0.002, df = 1, 26, p = 0.998). Note that each morph is likely to include multiple clones. (**e**,**f**)Reproductive rates of both the red and green morphs with and without ant attendance for an independent data set from (**a**–**d**). There is no difference between the rates of both the red and green (**e**; ANCOVA, *F* = 0.1881, df = 1,14, p = 0.671) but when ants have been removed the red morph showed a higher reproductive rate than the green morph under ant absence (**f**; ANCOVA, *F* = 30.7, df = 1, 18, p = 2.93e-5).

The results of experiment II (effects of ant attendance on the reproductive rate of each morph in mixed aphid colonies) are shown in Fig. 3a–d. In mixed aphid colonies, the reproductive rates of each morph were affected differently by ant attendance (Fig. 3). The reproductive rate of the red morph did not change in the presence or absence of ants (Fig. 3a; ANCOVA, *F* = 0.2049, df = 1, 26, p = 0.6546), whereas the reproductive rate of the green morph significantly increased when ants were in attendance (Fig. 3b; ANCOVA, *F* = 9.5709, df = 1, 26, p = 0.0047). Under the ant-excluded conditions, the red morphs increased more rapidly than the green morphs (Fig. 3c; ANCOVA, *F* = 3.094, df = 1, 26, p = 0.004), confirming the competitive superiority of the red morphs. However, this difference disappeared when ants were in attendance (Fig. 3d; ANCOVA, *F* = 2.1007, df = 1, 26, p = 0.15919). In addition, the results of another ant removal experiment from wild mixed colony showed the same conclusion, i.e, the reproductive rate is equalized under ant attendance (ANCOVA, F = 0.1881, df = 1,14, p = 0.671) but the red morph showed a higher reproductive rate than the green morph under ant absence (ANCOVA, F = 30.7, df = 1, 18, p = 2.93e-5; Fig. 3e,f). In conclusion, the attending ants equalized the reproductive rate of both the morphs such that the green morph became equally competitive with the red morph.

**Figure 4:**
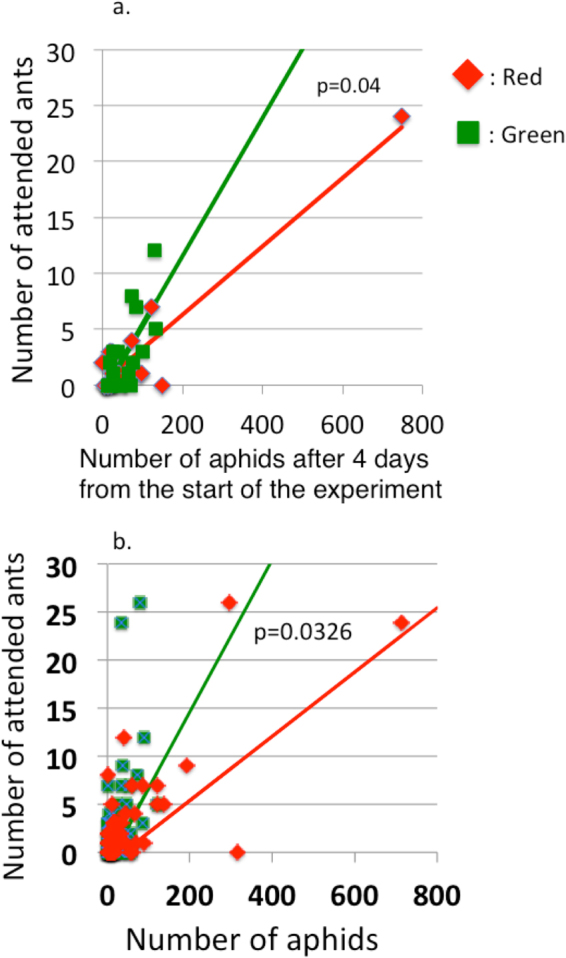
Relationships between the number of attending ants and the number of monoclonal aphids 4 days after the start of the experiment. (**a**) The slope of the regression line is significantly larger for the green morphs than for the red morphs (ANCOVA, t = 2.111, n = 17, P = 0.0432), indicating that more ants were attending to the green morphs when the number of aphids was equal. (**b**) An independent field samples showed the same result. In 51 mixed colonies collected in 2014, the numbers of attended ants have more steeper slope of regression to the number of the green morph than that to the red morph (ANCOVA, *F* = 4.6983, df = 1, 98, p = 0.0326). Both the results indicate that the green morph is more attractive to ants than the red one.

When wild ants were given a chance to select a red or a green monoclonal aphid colony, more ants per aphid selected the green colonies (Fig. 4). Another field data supported the same conclusion. Using 51 mixed colonies collected during late summer in 2014 it was shown that the numbers of attended ants has a more steeper regression slope (0.0793, t = 3.418, p = 0.0013) on the number of green morphs than that (0.03345, t = 7.639, p = 6.83e-10) on the number of red morph (for the difference, ANCOVA, F = 4.6983, df = 1, 98, p = 0.0326; Fig. 4b). These results indicate that the attended ant (*L. japonicus*) prefers the green morph over the red morph.

## Discussion

This study demonstrates that a mutualistic species actively maintains a polymorphism in its symbiont partners. The population-growth experiments of aphids showed that the red morph increased faster than the green morph, irrespective of variations in aphids or host plants (different clones) (Fig. 2), and that the red morph had a higher reproductive rate than the green morph. Accordingly, the red morph should dominate within a mixed colony on a shoot of the host plant. However, in the field, most aphid colonies are mixed and contain both red and green morphs^5^. Our results showed that attending ants equalize the reproductive rate of both the morphs to neutralize the competitive superiority of the red morph (Fig. 3a–f). As the two independent data sets reached the same result, the current conclusion should be more robust.

In ant-attended aphids, a tradeoff occurs between the quality of excreted honeydew and the fecundity of individuals^8^. This tradeoff may occur in *M. yomogicola* because the ants preferred the green morph over the red one (Fig. 4 and the above result for this issue), although the former showed lower reproductive rates than the latter without ant attendance (Fig. 2a,c). The attending ants likely improved the reproductive rate of the preferred green morph (Fig. 3b) to obtain a large amount of high-quality honeydew. Improved reproductive rates of ant-attended aphids have previously been reported^11,12^. In the current ant-aphid system, ants preferred mixed colonies with 65% green ants^5^, and the ants neutralized the competitive (clonal reproduction) inferiority of the green morph to maintain constant proportions of each morph (current results).

It is unclear why the ants do not remove the red morph from this symbiotic system. For attending ants, an increase in the less-valuable red morphs (with low-quality honeydew) should not be a preferable condition. In fact, *L. niger* (synonym of *L. japonicus*) selectively predates aphid individuals that excrete less honeydew in symbioses with other aphid species^13^. Ant workers (*L. japonicas*) should be able to discriminate the morphs of *M. yomogicola* because they increased the number of green morphs only in the mixed colonies (Fig. 3a–d). The workers also preferred a mixed colony with ca. 65% green morphs^5^. In a mixed colony, the ants only increased the reproductive rate of the green morphs. Ants are known to recognize opponents by the cuticular hydrocarbons of their body surface^14–18^. Therefore, in the current system, the recognition of green and red morphs by ants is suspected to be based on differences in the cuticular hydrocarbons on their body surface because ants selectively manipulate the reproductive rate of green morphs. In addition to their preference for the mixed colonies containing approximately 65% green morphs^5^, *L. japonicus* workers must have manipulated the aphid morphs to maintain the long-lasting coexistence of both morphs in every aphid colony. Therefore, the ants must have a reason for maintaining the red morph in their attended aphid colonies.

One possibility for maintaining the red morph is for the sexual reproduction of overwintering eggs (stem mothers) for the next year. Most of the *M. yomogicola* colonies disappear after inflorescence budding in host plants before the aphid colonies have been able to sexually produce the overwintering eggs. The red morphs may be better able to suppress the development of flower buds in host plants, allowing their colony to survive and produce sexuparae in late autumn. The pure green colony may fail to produce sexuparae for laying overwintering eggs. Therefore, the red morph may be important for maintaining the available aphid colonies as a honeydew resource for the attending ants in the next year. Because *L. japonicus* is a perennial species, the persistence of aphid colonies to the following year guarantees the presence of an available resource. In this case, the ants invest in a future benefit by sacrificing the present benefit. This behavior would maximize the lifetime reproduction of sexual alates (=fitness) of a *L. japonicus* colony. This interesting hypothesis is currently being tested, and if supported, each of the three participants in this symbiotic system would receive fitness benefits from the long-lasting coexistence with genetically heterogeneous contributors.

Few studies have focused on the effects of mutualism in community ecology^19^. Mutualism may contribute to the origin and maintenance of biodiversity^20,21^. For example, the extreme biodiversity of trees in tropical rainforests may be mediated by repeated speciation of tree-symbiont (animal seed dispersers) systems during glacial periods^22^. In this report, we discovered another case in which symbiosis actively maintains polymorphic diversity in aphids.

## Methods

### Study organisms

*Macrosiphoniella yomogicola* is an aphid that colonizes mugwort (*Artemisia vulgaris*). Several color morphs occur in this aphid^5^, although in our study area (the campus of Hokkaido University at Sapporo, Hokkaido, Japan), two morphs (red or green) are usually found. In early May at Sapporo, a stem mother hatches from an overwintered egg that was laid by sexual reproduction the previous autumn. She produces clonal offspring by asexual reproduction, and her offspring continue asexual reproduction until autumn. During this period, the aphids inherit their body color. Although *M. yomogicola* colonies are always attended by several species of ant^6^, we only used colonies with the attendant-ant *Lasius japonicus* because most aphid colonies in our study area are attended by this species.

### Experiment I: Difference in reproductive rates between the two color morphs

I-1. In May 2014, we reared one red adult and one green adult on separate mugwort shoots of the same clone plant without ants in the greenhouse of our facility. Each shoot was covered by a nylon mesh (30 × 20 cm) to protect the colonies from predators. The number of aphids was recorded once every 2 or 3 days. This process was repeated for five different clones of both red and green morphs on a single host plant clone, and the results were averaged for each morph (Fig. 2a).

I-2. From June to September 2014, five red and five green clones were reared independently on 10 shoots each of the same host plant clone without ants at the same location as experiment I-1. Predators were excluded using the same method as in I-1, and the number of aphids was recorded once every 3 days (Fig. 2b).

I-3. From May to August 2016, a red clone and a green clone were each reared on a shoot of the same host plant clone with ants, resulting in a large clonal colony. From the clone pool of each morph, 3 adult aphids were transferred to one of 5 shoots of 5 host plant clones. We recorded the number of aphids on each shoot without ants once every 3 days (Fig. 2c). Predator exclusion was conducted in the same way as in the above two experiments.

### Experiment II: Equalization of the reproductive rate of both the morphs by ant attendance

2-1. In June 2014, thirty host plants were parasitized by mixed aphid colonies (containing both morphs), and 15 of the thirty plants were attended by ants by connecting each plant to a randomly selected mugwort that had an ant-attended aphid colony in the field. One pair of shoots was also selected randomly. One shoot remained with ant attendance, and the other shoot was rubbed with a sticky liquid (Tanglefoot®) at its base to remove attending ants. The latter shoot was covered with nylon mesh to protect it from predators. The number of each morph was recorded for all shoots before the experiment and then was recounted 3 days later (Fig. 3). The two sets of numbers were plotted to separate color morphs, and the slopes of the liner regressions were compared by treatment to determine whether the population growth of both morphs differed with and without ant attendance.

2-2. At 23 July and 24 September 2016, we randomly selected 14 and 8 mugwort shoots that were paratisized by mixed aphid colonies in the study area, respectively. For both the shoot groups the numbers of both the morphs were recorded at the start day (the above days). For the former 14 shoots, we removed attended ants by rubbing Tanglefoot® at the base of the mugworts’ stem, and the shoots were covered by a nylon-mesh bug to prevent the aphid colonies from predations. After 3days, the shoots were transported to the laboratory within the bugs and the numbers of both the morphs were counted under a byocular microscope (Olympus 243702 Olympus, Tokyo, Japan). For the later 8 shoots, the shoots were remained under ant attendance, and the numbers of both the morphs were recorded again at 3 days after. For both treatments, we regressed the numbers of each morph at the end of the experiment on the numbers at the start. Then, the slopes of the regression line of each morph were compared by ANCOVA for each data set.

### Experiment III: Ant preference on each morph

3-1. From June to September, 19 clonal shoots were prepared in a plastic pod (15 × 20 cm), and each shoot was parasitized with a clone of a color morph. In addition, 19 pairs of pods were prepared with monoclonal colonies of each color morph. Nineteen wild host plants with aphid colonies that were parasitized with ants were selected randomly, and a pair of pods with different color morphs was connected to a wild host plant using a long thin bamboo stick to enable the attending ants of the wild host plant to reach the connected reared monoclonal colonies. The numbers of aphids and attending ants were counted on each shoot 4 days later. In one pair, a green morph had been extirpated within 4 days; therefore, the data for this pair were removed from the analysis. In addition, in another pair, a green colony attracted too few ants and did not have a normal distribution of symbiont ants and aphids (P = 0.0001). Because the data for this pair were abnormal, they were also removed from the analysis. One colony included far more red morphs (749 individuals) than the other colonies (1–149 individuals), although the aphids/ants ratio (for which we calculated the linear regression) of this colony (0.03204) was within the lower 95% limits (0.00154) of the F distribution (df = 1,1). Thus, this colony was not considered an abnormal statistical outlier, and these data were included in the analysis. As a result, we examined the ants’ preference using 17 pairs of pods (Fig. 4).

3-2. We analyzed another field samples to confirm the ant preference to the green morph. During late summer in 2014, 51 mixed colonies including both the morphs were sampled from the study area with attending ants. The numbers of attending-ants, the red and green aphids were recorded for each shoot. The numbers of ants was regressed on each the number of reds or greens, and the slopes was compared by ANCOVA.

### Statistics

All the statistics were performed by R ver. 3.2.1.
